# Correction to “Inhibition of tripartite motif containing 26 inhibits non‐small cell lung cancer cell growth”

**DOI:** 10.1002/kjm2.12751

**Published:** 2023-09-12

Xia YC, Zha JH. Inhibition of tripartite motif containing 26 inhibits non‐small cell lung cancer cell growth. Kaohsiung J Med Sci. 2021;37(5):440–441. https://doi.org/10.1002/kjm2.12330

There is an error in Figure 1. In Figure 1d, The a549 con and si‐2 were the same image.

**FIGURE 1:**
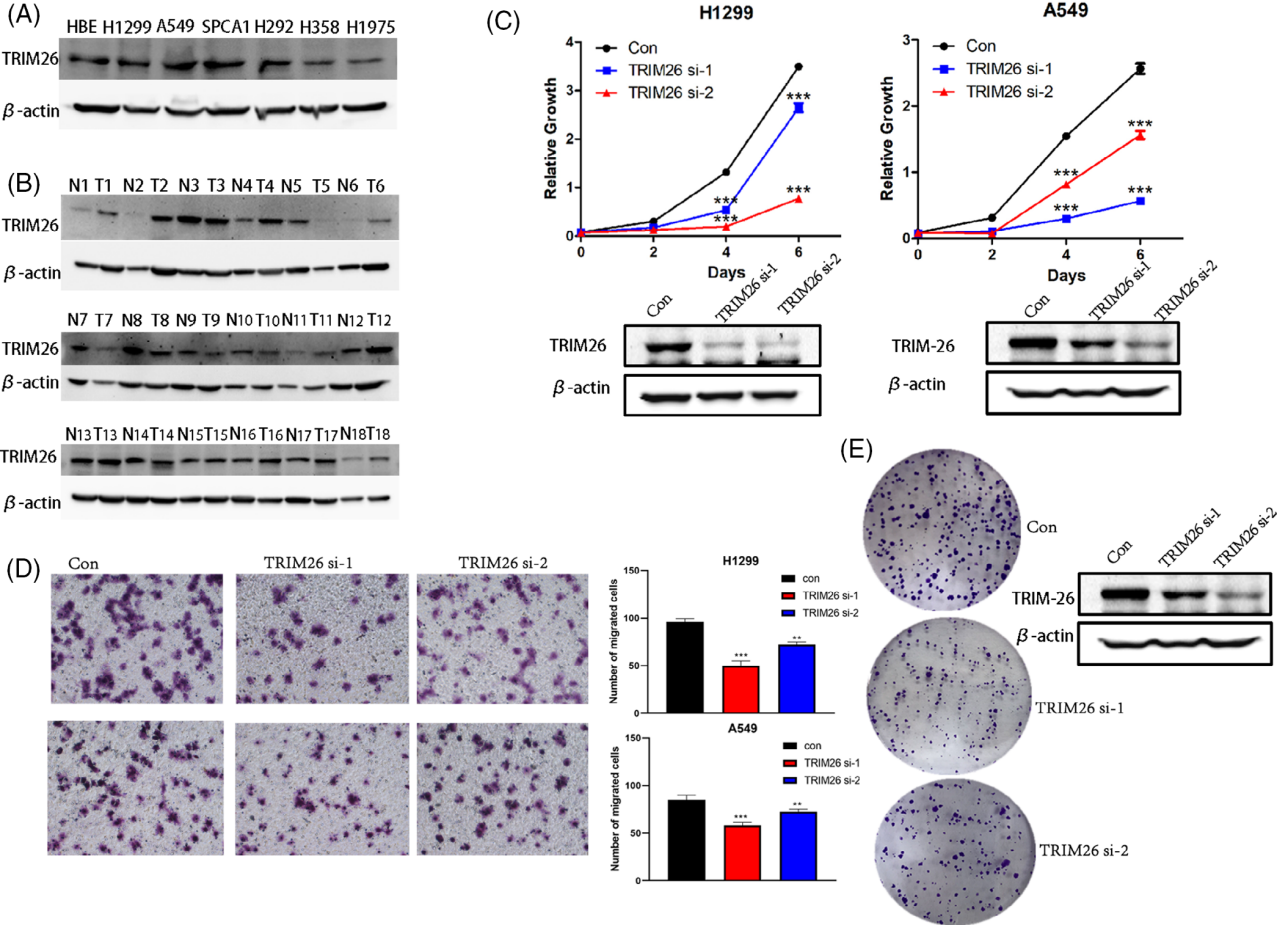
Knockdown of TRIM26 inhibits cell growth and immigration in NSCLC cells. (A) NSCLC cell lines were prepared for immuno‐blotting against TRIM26 and β‐actin. (B) NSCLC primary tumor tissues were collected for immuno‐blotting against TRIM26 and β‐actin. A549 and H1299 cells were infected with TRIM26 RNA interference or TRIM26 control, respectively. After culturing cells then treatment with trypsin digestion, some of the cells were counted out for clonal colony formation assays and another part of cells for MTT assays at same time. (C) MTT were performed to evaluate the cell viability, **p < 0.05. (D) Transwell chamber were used to measure cell migration, respectively. (E) Colony formation were measured, respectively.

In Figure 1c and e the wb of A549 is same because we transfected with si‐1 si‐2 and control into A549 cells, after treatment with trypsin digestion, some of the cells were counted out for clonal colony formation assays and another part for MTT assays, we then extracted remaining cells protein for wb assay. So the same wb images were used for 1c and 1e of a549 to illustrate the knockdown efficiency.

Below is the correct figure and figure legend.

This correction does not affect the results or conclusions drawn from the data in any way.

